# Fast-acting and injectable cryoneurolysis device

**DOI:** 10.1038/s41598-022-24178-6

**Published:** 2022-11-18

**Authors:** Sara Moradi Tuchayi, Ying Wang, Alla Khodorova, Isaac J. Pence, Anat Stemmer-Rachamimov, Conor L. Evans, R. Rox Anderson, Lilit Garibyan

**Affiliations:** 1 Wellman Center for Photomedicine, Massachusetts General Hospital, 50 Blossom Street-Thier 2, Boston, MA 02114 USA; 2 Massachusetts General Hospital, Department of Dermatology, Harvard Medical School, Boston, USA; 3 Department of Pathology, Harvard Medical School, Boston, MA USA

**Keywords:** Trauma, Pain, Chronic pain

## Abstract

Cryoneurolysis is an opioid-sparing therapy for long-lasting and reversible reduction of pain. We developed a nerve-selective method for cryoneurolysis by local injection of ice-slurry (− 5 to − 6 °C) that induced decrease in nocifensive response starting from about a week after treatment and lasting up to 8 weeks. In this study, we test the hypothesis that injection of colder slurry leads to faster onset of analgesia. Colder slurry (− 9ºC) was injected around the rat sciatic nerve to induce cryoneurolysis. Hematoxylin and Eosin (H&E) staining was used to examine histologic effects on surrounding tissues. Coherent anti-Stokes Raman scattering (CARS) microscopy was used to study effects on myelin sheaths. Functional tests were used to assess changes in sensory and motor function in the treated hind paw. No inflammation or scarring was detected in surrounding skin and muscle tissues at day 7 post slurry injection. Functional tests showed rapid onset reduction in mechanical pain sensitivity starting from day 1 and lasting up to day 98. CARS imaging demonstrated disintegration of myelin sheaths post treatment followed by complete recovery of nerve structure by day 140. In this study we showed that colder slurry (− 9 °C) produces more rapid onset and longer duration of analgesia, while remaining nerve-selective

## Introduction

Cryoneurolysis is an opioid-sparing therapy for long-lasting and reversible reduction of pain. Current cryoneurolysis devices use invasive cryoprobes with very low-temperatures (− 60 °C and below) to freeze target peripheral nerves^1–3^. Cryoprobe placement directly on the nerve is critical to achieve good outcomes without damaging surrounding tissue^4^. To increase success and to avoid non-selective tissue damage, most treatments require surgical exposure of the target nerve or use of image guidance, making the procedure invasive, time-consuming, and expensive^4^. In addition, use of conventional cryoneurolysis methods, similar to other neuro-ablative procedures is further limited due to the common side effect of dysesthesia which presents as pain, burning and tingling in the treated area^5–9^. The use of extremely low temperatures in current cryoneurolysis devices has been postulated to contribute to dysesthesia post cryoneurolysis.

We previously described a neural-selective injectable method for cryoneurolysis using sterile ice slurry, that overcomes the limitations of current cryoneurolysis devices^10^. Injection of slurry was demonstrated to be nerve-selective without any damage to surrounding muscle and skin tissues^10^. We showed that a single local injection of slurry (at − 5 to − 6 °C) around the rat sciatic nerve, using a standard syringe and needle, leads to long-lasting reduction in mechanical pain^10^. In addition to ease of use and long-lasting effects, slurry injection did not cause dysesthesia, due to its ability to selectively reduce myelinated fibers without affecting unmyelinated C fibers^11^. The selective targeting of myelinated fibers and sparing unmyelinated C fibers resulted in decreased mechanical pain sensitivity while preserving thermal pain sensitivity, as unmyelinated C fibers are the main category of nerve fibers conducting thermal pain^12^. Our work has also demonstrated that repeated treatments with injectable ice slurry are nerve-selective, recover completely, and appear to be safe^13^, which will be important for patients with chronic pain as they will require repeated injections.

Ice slurry injection at − 5 to − 6 °C significantly decreased nocifensive response starting from about a week after treatment, and lasting up to 8 weeks when compared to baseline^10,11^. While the long duration of the slurry induced analgesia is desirable, its late onset could limit clinical applications for pain reduction. In this study, we test the hypothesis that injection of colder slurry leads to faster onset of analgesia. We also investigate safety and efficacy of colder slurry for inducing nerve selective cryoneurolysis in a rat sciatic nerve model.

## Methods

### Animals

Animal studies were approved by Massachusetts General Hospital Institutional Animal Care and Use Committee (IACUC). All experiments were performed in accordance with the Guide for the Care and Use of Laboratory Animals published by the US National Institutes of Health. Authors complied with the ARRIVE guidelines. Adult male Sprague–Dawley rats 200–250 g and 7–8 weeks old were purchased from the Charles River Laboratories (Wilmington, MA). Animals were housed under pathogen-free conditions in an animal facility at the Massachusetts General Hospital in accordance with animal care regulations. Twenty-five animals were used in this study. Carbon dioxide overdose followed by bilateral thoracotomy as secondary physical method was used as the method of euthanization.

### Cooling procedure

Ice Slurry composed of normal saline with 15% glycerol was made to a slurry temperature of -9 °C, with a prototype device (Sage Product Development Inc, Foxborough MA). Rats were randomly assigned to test or control groups and injected with 15 ml of slurry or the room temperature control solution (melted slurry) around the sciatic nerve using 15-gauge needle as previously described^10^.

### Mechanical nocifensive test (von Frey assay)

Paw Withdrawal Frequency (PWF) as measure of sensitivity to punctate mechanical stimulation was determined using calibrated von Frey hairs as described previously^10,14^.

### Thermal test

The sensitivity of the plantar paw to noxious radiant heat was determined using Hargreaves Apparatus (Ugo Basile, Varese, Italy). Paw withdrawal latency (PWL) in response to heat stimulation was determined by the amount of time that animal takes to move the hind limb after the start of heat emission as described previously ^10,15^.

### Toe spread test

Physiologic toe spread reflex was assessed using a modified toe spread test to identify changes in muscle function after treatment with slurry as described previously ^10,16^.

### Walking test

Motor function was also assessed by walking behavior as previously described ^10,17^.

### Coherent anti-Stokes Raman scattering (CARS)

CARS microscopy was used to assess myelin and nerve structure as previously described^10,11^. Briefly, the CARS microscope used a confocal microscope (Olympus FV1000, Center Valley, PA) modified with a dual output femtosecond pulsed laser system (Spectra-Physics Insight DeepSee, Santa Clara, CA) to generate Raman signal from myelin sheath lipids via CH bond vibrational modes. Depth stacks were acquired from the surface of the nerve to 20 µm within the tissue at 1-µm intervals, resulting in 21-frame image stacks. The volumetric image sets were projected to 2D images, using false color to denote depth within the stack: cyan features are most superficial, while red features are deeper within the tissue. A series of three such images were acquired from each sample. The corrected correlation parameter (CCP) was extracted to assess myelin structure following the methodology described previously^10,11,18^. CCP corresponds to orderly structure of myelin sheaths.

### Histology

Samples of surrounding skin and muscle at the injection site were processed and embedded in paraffin, and sectioned at 5 μm. Sections were then deparaffinized, and stained with hematoxylin and eosin (H&E). A board-certified neuropathologist blindly assessed biopsy samples for histologic changes.

### Statistical analysis

Statistical analysis was performed using Prism 9 (GraphPad Software, Inc., La Jolla, CA). Sample size selection was based on our previous experiments with this rat model and tests. Rats were randomly assigned to test or control groups. Data were tested for normality using the Shapiro–Wilk normality test and non-parametric tests were used where an outcome measure did not follow the normal distribution. Friedman test followed by Dunn’s multiple comparisons test was used as the test of significance for paw withdrawal frequency, paw withdrawal latency, toe spread and walking scores. Ordinary one-way ANOVA followed by Tukey's multiple comparisons test was used as the test of significance for corrected correlation parameter (CCP) index. *P* < 0.05 was considered significant. Graphs show mean + SD or median with interquartile range as noted.

## Results

**Figure 1:**
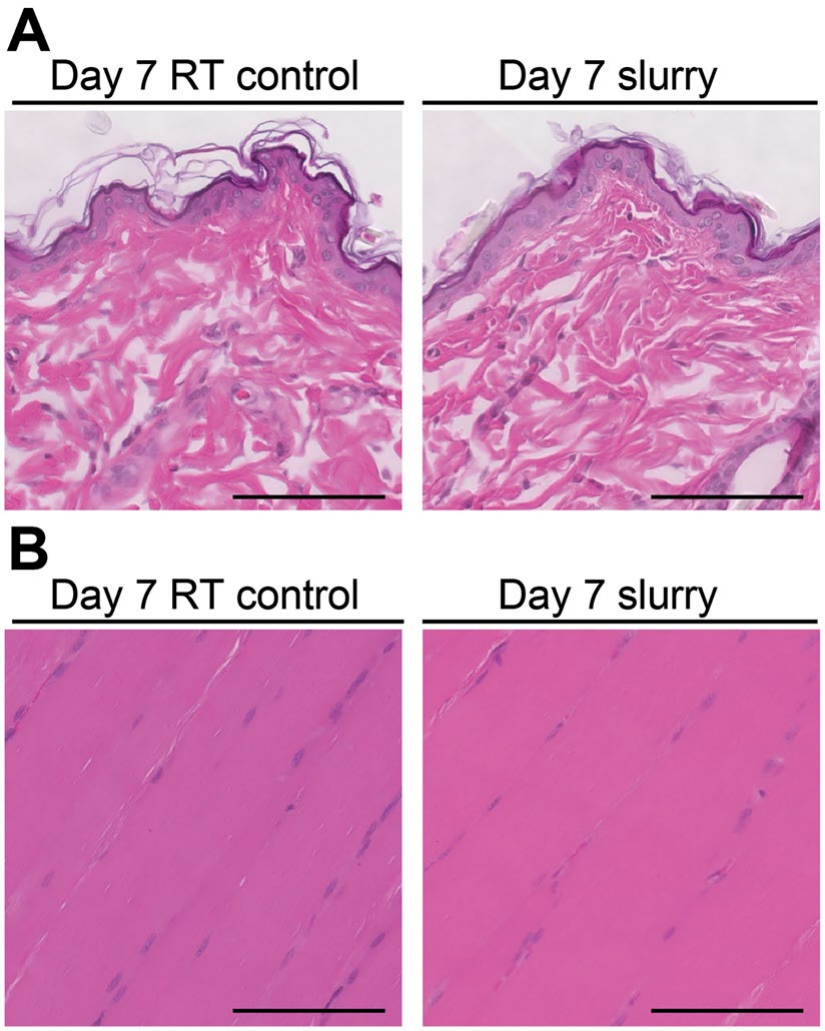
Slurry is selective to nerve tissue with no damage to surrounding skin or muscle tissue. Representative images of (**A**) skin, and (**B**) muscle tissue at the injection site stained with H&E at 7 days after injection of slurry; scale bar, 100 μm; RT, room temperature.

Injection of − 9 °C slurry around sciatic nerve in rats was well tolerated without any systemic adverse effects, or local nonselective damage to the surrounding skin or muscle tissue (Fig. 1A, B). H&E stained sections from samples treated with − 9 °C slurry or the room temperature solution collected at day 7 post injection showed normal histology without any damage in the surrounding skin, and muscle tissue (Fig. 1A, B).

**Figure 2:**
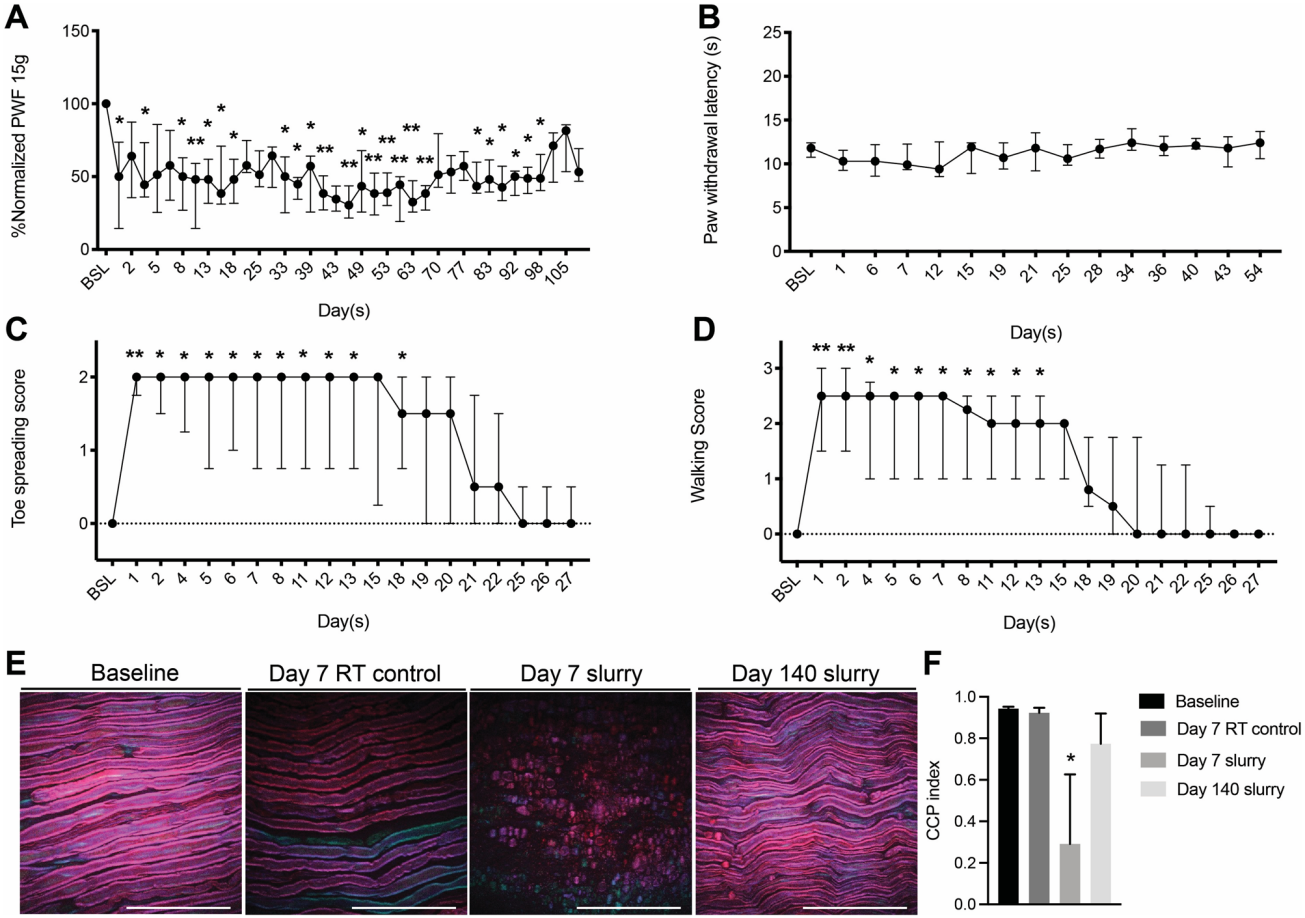
Slurry shows rapid onset reduction in nerve function (**A**) Paw withdrawal frequency in response to stimulation with 15 g VFH force at different time points post slurry injection. (**B**) Effects of slurry injection on paw withdrawal latency in response to heat stimulation in treated hind limbs. (**C**) Toe spread and (**D**) walking scores of hind limbs treated with slurry. Data are presented as median with interquartile range. n = 9; * *P* < 0.05, ** *P* < 0.001 compared to baseline values by Friedman test followed by Dunn’s multiple comparisons test. (**E**) CARS microscopy images show myelin and nerve structure in sciatic nerve samples after injection of slurry and room temperature control solution. (**F**) Graph shows CCP index after injection of slurry and room temperature control solution. Data are presented as mean ± SD. n = 4 per group; * *P* < 0.0001 compared to baseline values by Ordinary one-way ANOVA followed by Tukey's multiple comparisons test; RT: room temperature.

Slurry induced significant decrease in mechanical pain sensitivity in comparison to baseline starting from day 1 post slurry injection (median [interquartile range]: %50.00 [%14.50, %73.64] at day 1 vs %100.00 [%100.00, %100.00] at baseline, *P* < 0.05) (Fig. 2A). This rapid onset decrease in mechanical pain sensitivity lasted up to day 98 (Fig. 2A). The thermal heat pain sensation was not affected (Fig. 2B), as previously seen with − 5 to − 6 °C slurry. Motor function was also affected (Fig. 2C and D), but recovered fully by day 25 consistent with previous data^10,11^. Injection of the room temperature control solution did not cause any significant changes in mechanical and thermal heat pain sensitivity, or motor test results in comparison to the baseline (Supplementary Fig. 1).

The effect of colder slurry on myelin morphology and nerve structure was assessed using CARS microscopy in treatment and room temperature control groups. CARS microscopy of sciatic nerves 7 days after slurry injection showed structural changes; there was significant decrease in Corrected Correlation Parameter (CCP) value (0.29 ± 0.34 at day 7 vs 0.94 ± 0.01 at baseline, *P* < 0.0001), indicating disruption of nerve and myelin structure (Fig. 2E and F). CCP values returned to normal range at day 140 post treatment (0.77 ± 0.15 at day 140 vs 0.94 ± 0.01 at baseline, *P* = 0.1560), indicating nerve and myelin structure recovery. Injection of room temperature control solution did not cause notable changes in the nerve (0.92 ± 0.025 at day 7 post injection of room temperature control solution vs 0.94 ± 0.01 at baseline, *P* = 0.9947).

## Discussion

In this study we showed that colder slurry (− 9 °C) produces more rapid onset and longer duration of analgesia when compared to baseline mechanical sensitivity values. The − 9 °C slurry remained nerve-selective as there was no damage to surround skin and muscle tissues. The − 9 °C slurry remained nerve-selective as there was no damage to surround skin and muscle tissues. We have previously shown that injection of − 5 to − 6 °C slurry around the rat sciatic nerve leads to a significantly decreased mechanical pain when compared to baseline values, starting from about a week after treatment and lasting for about 8 weeks, with full^10,11^. In this study we examined the effects of injection of the colder slurry (− 9 °C) on mechanical pain sensitivity in comparison to baseline sensitivity values (Fig. 2A). Functional exams showed a significant reduction in mechanical pain sensitivity starting from day 1 post treatment and lasting up to day 98 (Fig. 2A). . Based on comparison of mechanical pain sensitivity values at various time points after treatment to that of baseline values, the − 9 °C slurry treatment shows significant reduction as early as day 1. The explanation for this more rapid onset of analgesia could be multifactorial. We have previously shown that total ice content and the initial temperature of the ice slurry determine the extent of its effects on adipose tissue and the rate of cooling^19,20^. Due to different composition of glycerol (10% vs 15% respectively), the − 6 °C slurry and − 9 °C slurry have similar total ice content of about 44%. Given the same ice content and volume of ice-slurry used in this study, the rapid onset of analgesia could be explained by increased rate of cooling with colder ice-slurry that has lower initial temperature. The increased rate of cooling with colder slurry might cause more rapid disruption of myelin and therefore more rapid onset of analgesia.

The increased rate of tissue cooling likely leads to more ice crystal formation and phase transition in myelin lipids, which is more destructive. It has been reported that more rapid cooling of cells leads to more intracellular ice formation which is more destructive for cells^21^.

Another explanation could be that slightly higher glycerol concentration in the colder (− 9 °C) ice slurry creates a more hyperosmolar environment around the nerve which could affect nerve activity and response to cooling. The lethality of ice crystals formed during cooling depends on the osmotic composition of solution in which the tissue is cooled^22^. During cooling the plasma membrane of cells is damaged at a critical gradient in osmotic pressure across membrane, and exposure of cells to osmotic stressors can induce membrane damage and intracellular freezing^23^. Thus, it is possible that the hyperosmolar composition of the − 9 °C slurry is able to induce more disruption of the target nerve resulting in more rapid onset of analgesia.

More work needs to be done to determine the exact mechanism of rapid onset analgesia with colder slurry. The longer duration of analgesia with the colder slurry is consistent with previously reported finding that colder temperatures caused longer duration of sensory loss with cryoneurolysis^24^.

Importantly, rapid onset and longer duration of analgesia is desirable to the extent that slurry does not lose its selective effects on myelinated nerve fibers. Although analgesia induced by current cryoneurolysis devices that use cryoprobes with very low-temperatures (− 60 °C and below) has rapid onset and is long-lasting, it is accompanied by dysesthesia side effect that happens as a result of loss of unmyelinated C fibers^11^. Lack of any changes in noxious thermal sensitivity with -9ºC slurry, similar to the − 5 to − 6 °C slurry, hints at sparing of unmyelinated C fibers hence will likely avoid the dysesthesia side effect.

CARS imaging and histological analysis showed that the colder slurry, similar to − 5 °C to − 6 °C slurry, induced nerve-selective cryoneurolysis targeting myelin sheaths without any damage to surrounding muscle and skin tissues. Similar to the − 5 to − 6 °C slurry, − 9 °C treated nerves showed full recovery of function and structure.

Rapid onset of analgesia combined with prolonged duration of effects, nerve-selective targeting, full recovery, and the familiarity and ease of the injection procedure make ice slurry injection an effective and more accessible method of cryoneurolysis. The more rapid onset of analgesia after injection of colder slurry, potentially makes this therapy useful for non-opioid control of pain after surgery or trauma, while the long duration of analgesia potentially makes this therapy useful for reducing chronic pain.

## Supplementary Information

Supplementary Information 1.

## Data Availability

All data are available in the main text or supplementary materials.
