# Supplementary material for: Fast-acting and injectable cryoneurolysis device
Source: Sci Rep. 2022 Nov 18;12:19891. doi: 10.1038/s41598-022-24178-6 (PMC9674623; doi:10.1038/s41598-022-24178-6)
Supplement: Supplementary file 1 — Supplementary Information 1. [file 41598_2022_24178_MOESM1_ESM.docx]

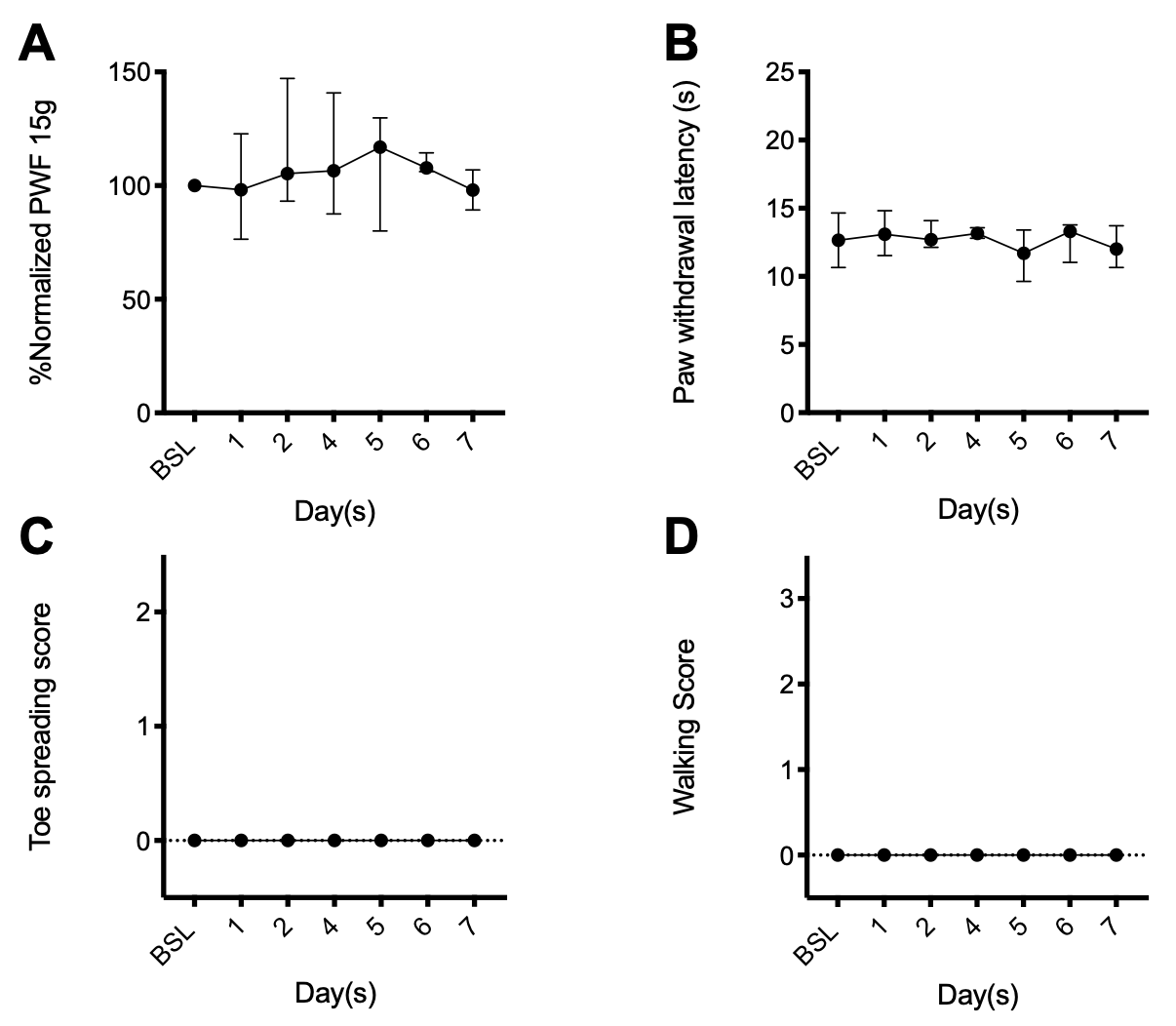


Supplementary Figure 1. Injection of room temperature control solution does not affect nerve function (**A**) Paw withdrawal frequency in response to stimulation with 15g VFH force during the first 7 days post injection with room temperature control solution. (**B**) Paw withdrawal latency in response to heat stimulation in hind limbs injected with room temperature control solution. (**C**) Toe spread and (**D**) walking scores of hind limbs injected with room temperature control solution. Data are presented as median with interquartile range. n=4.
